# Downregulation of Steroid Receptor Coactivator-2 Modulates Estrogen-Responsive Genes and Stimulates Proliferation of MCF-7 Breast Cancer Cells

**DOI:** 10.1371/journal.pone.0070096

**Published:** 2013-07-30

**Authors:** Ingvild S. Fenne, Thomas Helland, Marianne H. Flågeng, Simon N. Dankel, Gunnar Mellgren, Jørn V. Sagen

**Affiliations:** 1 Department of Clinical Science, University of Bergen, Bergen, Norway; 2 Hormone Laboratory, Haukeland University Hospital, Bergen, Norway; Karolinska Institutet, Sweden

## Abstract

The p160/Steroid Receptor Coactivators SRC-1, SRC-2/GRIP1, and SRC-3/AIB1 are important regulators of Estrogen Receptor alpha (ERα) activity. However, whereas the functions of SRC-1 and SRC-3 in breast tumourigenesis have been extensively studied, little is known about the role of SRC-2. Previously, we reported that activation of the cAMP-dependent protein kinase, PKA, facilitates ubiquitination and proteasomal degradation of SRC-2 which in turn leads to inhibition of SRC-2-coactivation of ERα and changed expression of the ERα target gene, pS2. Here we have characterized the global program of transcription in SRC-2-depleted MCF-7 breast cancer cells using short-hairpin RNA technology, and in MCF-7 cells exposed to PKA activating agents. In order to identify genes that may be regulated through PKA-induced downregulation of SRC-2, overlapping transcriptional targets in response to the respective treatments were characterized. Interestingly, we observed decreased expression of several breast cancer tumour suppressor genes (e.g., *TAGLN*, *EGR1*, *BCL11b*, *CAV1*) in response to both SRC-2 knockdown and PKA activation, whereas the expression of a number of other genes implicated in cancer progression (e.g., *RET*, *BCAS1*, *TFF3*, *CXCR4*, *ADM*) was increased. In line with this, knockdown of SRC-2 also stimulated proliferation of MCF-7 cells. Together, these results suggest that SRC-2 may have an antiproliferative function in breast cancer cells.

## Introduction

The Steroid Receptor Coactivator (SRC) family, also known as p160 proteins, consist of the three members, SRC-1 (NCOA1) [1], SRC-2 (NCOA2/GRIP1/TIF2) [2], [3], and SRC-3 (NCOA3/AIB1/ACTR/RAC-3/pCIP/TRAM-1) [4]–[8]. Even though SRC-2 is functionally and structurally related to the other two SRC members, knockout studies in mice have shown that SRC-2 plays distinct functional roles in fertility and ductal branching in mammary gland [9], [10], glucose- and lipid metabolism [11], [12], regulation of bone mass [13], cardiac function [14] and progesterone-dependent cell cycle and immunity [15]. The SRC-specific functions are believed to be due to their tissue-specific expression levels, different affinities for various NRs, competition between NRs to recruit SRCs and between SRCs themselves for binding to NRs, and different post-translational modifications (PTMs) that regulate their protein levels and activity [16]–[18]. There is also evidence that SRC-2, in contrast to SRC-1 and SRC-3, has repressive effects on specific ER- and glucocorticoid receptor (GR) target genes in the presence of their respective ligands [19]–[22]. While the roles of SRC-1 and SRC-3 have been extensively studied in breast cancer, less is known about the function of SRC-2 in regulating genes involved in breast cancer progression. In contrast to SRC-2, SRC-1 and SRC-3 are frequently overexpressed in a subset of breast cancers, and correlate with a more aggressive tumour phenotype and poor prognosis and resistance to endocrine treatment [4], [23]–[28]. Even though depletion of SRC-2 in MCF-7 cells has been shown to decrease estrogen-dependent ERα transactivation function, loss of SRC-2 does not seem to affect estrogen-dependent proliferation of MCF-7 cells [29]. This is in contrast to studies of SRC-3-depleted MCF-7 cells in which the estrogen-dependent proliferation of the cells was significantly reduced [29]–[32]. Molecular studies of each SRC in MCF-7 breast cancer cell suggest that the SRCs exhibit differential regulation of endogenous ER-target genes, indicating specific contributions of each SRC member to promote breast cancer [17], [29], [33], [34]. In addition, distinct PTMs of the SRC members play a crucial role in controlling their intracellular levels and functions, which may have significant impact on breast carcinogenesis and response to endocrine treatment in breast cancer patients [18]. In previous studies we have demonstrated that activation of the cAMP-dependent protein kinase (PKA) signalling pathway targets SRC-2 coactivator function through its ubiquitination and proteasomal degradation. This in turn inhibits SRC-2-mediated coactivation of ERα and modulates transcription of the ERα target gene pS2 [35]–[37].

In the present study, our aim was to characterize the role of SRC-2 on global expression of genes in MCF-7 breast cancer cells. We also wanted to explore the effects of PKA-induced degradation of SRC-2 on the expression of genes involved in breast tumourigenesis. Interestingly, our data suggest that SRC-2 is important for the expression of various ER-target genes linked to breast cancer progression, including specific oncogenes and tumour suppressor genes. A subset of these genes was also found to be modulated through PKA-induced downregulation of SRC-2. Moreover, proliferation data suggested that knockdown (KD) of SRC-2 stimulated proliferation of MCF-7 cells. Taken together, our results suggest that SRC-2 play an important role in regulating expression of a subset of ER-target genes involved in proliferation of MCF-7 cells.

## Materials and Methods

### Cell Cultures

MCF-7 human breast adenocarcinoma cells and Human embryonic kidney 293T (HEK 293T) cells (ATCC) were grown at 37 °C and 5% CO_2_, in Dulbecco’s modified Eagle’s medium (DMEM) (Cambrex, Verviers, Belgium) supplemented with 4,5 g/liter glucose, 2 mM L-glutamine, 10% foetal bovine serum (FBS), 100 units penicillin, 100 µg streptomycin. The MCF-7 cell medium also contained 1 µM insulin. For experiments MCF-7 cells were seeded in phenol red-free DMEM supplemented with 5% charcoal-stripped FBS.

### Short Hairpin RNA (shRNA) lentiviral Transduction

Five individual lentiviral pLKO.1-puro short hairpin (sh) RNA plasmids targeting different sequences on SRC-2/GRIP1 or SRC-3/AIB1 mRNA and a pLKO.1-puro empty vector control were purchased from Sigma Mission® RNAi (Sigma). To produce lentiviral stocks each plasmid was cotransfected with the lentiviral packaging plasmids psPAX2 and pMD2G into individual plates with HEK 293T cells using SuperFect (QIAGEN, Valencia, CA). The virus containing cell culture supernatants from each culture were collected 48 h after transfection and used for lentiviral transduction of individual MCF-7 cells cultures in the presence of 10 µg/ml µg polybrene per ml virus supernatant. Two days after infection 1 µg/ml puromycin was added in order to select for infected cells. The puromycin selections were maintained for three weeks to obtain cells containing stably integrated shRNA.

### RNA Extraction and Quantitative Real-time PCR

Total RNA from MCF-7 cells was extracted using RNeasy Mini kit (Qiagen, CA). 1 µg RNA was reverse transcribed using the cDNA synthesis kit (Roche Basel, Switzerland). The real time quantitative reverse transcription (qRT)-PCR analyses were carried out using the LightCycler® RNA Master SYBR Green I kit in a LightCycler rapid thermal cycler system (Roche, Basel, Switzerland). The mRNA expression levels of target genes were quantified relative to the housekeeping gene TATA-binding protein (TBP). The primer sequences are provided in the Materials and Methods Supporting Information (Table S1).

### Microarray Preparation of Samples

The shRNA-expressing MCF-7 cells were grown for three days in phenol red-free DMEM with 5% charcoal-stripped FBS and 10 nM 17β-estradiol. RNA was extracted from five independent plates of cells (replicates) expressing the pLKO.1-puro empty shRNA vector (Control shRNA), from five replicates of SRC-2 shRNA expressing cells (SRC-2 shRNA), and from five replicates of Control shRNA cells treated with 150 µM 8-parachlorophenylthio-cAMP (8-CPT-cAMP), 50 µM 3-isobutyl-1-methylxanthine (IBMX), and 10 µM forskolin for 24 hours. Sample preparations were balanced and randomized in each step of lysate collection, RNA extraction and labelling, and microarray hybridization. Microarray was performed using the Illumina HumanRef-8 v 3.0 Expression BeadChips.

### Microarray Analysis Using the Illumina Iscan System

400 ng of total RNA from each cell sample (three biological groups, five samples within each group, 15 samples total) was biotin-labelled and amplified using the Illumina TotalPrep RNA amplification kit (version 0606, Ambion®, USA) and the Eppendorf Mastercycler (Eppendorf®, Germany). Quality and quantity measurement of the biotin-labelled cRNA were performed using the Agilent 2100 Bioanalyzer and the NanoDrop® ND-1000. 750 ng of cRNA was thereafter hybridized to the HumanRef-8 v.3.0 Expression Bead Chips containing gene-specific probes at 58°C for 17 hours. The HumanRef-8 v.3.0 Expression BeadChip targets approximately 24500 genes derived from human genes in NCBI RefSeq database. The hybridization was performed according to the Whole – Genome Gene Expression Direct Hybridization Assay Guide (Illumina Inc.). The fluorescence of the biotin-labelled cRNA was measured using the iScan Reader (Illumina Inc.). Analyses were performed at the Norwegian Microarray Consortium (NMC) Core Facility, University of Bergen, Norway.

### Microarray Data Extraction and Analysis

The raw data from the microarray was imported into GenomeStudio Data Analysis Software (Illumina, Inc.) for quality controls. The control probes were then removed and a text file containing the signal and detection p-values per probe for all samples was created and imported to J-Express Pro software version 2009 (MolMine, Norway). Quality controls and analyses in J-Express were performed on quantile normalized and logarithmically transformed (base 2) signal intensity values [38]. Correspondence Analysis (CA) and hierarchical clustering with Pearson Correlation as a distance measure were applied to study global trends in the data and search for outliers within the sample groups [39]. Differentially expressed genes were detected through the Significance Analysis of Microarray (SAM) [40], and defined by fold change of at least 1.3 and q-value = 0. To search for over-represented functional categories among the differentially expressed genes, Protein ANalysis THrough Evolutionary Relationships (PANTHER) (version 7, http://www.pantherdb.org) was used to organize differentially expressed genes in categories representing biological functions and molecular functions. The Bonferroni correction for multiple testing was used to calculate p-values for the over-represented categories.

### Western Blotting

Procedures for Western blotting are previously described (Hoang et al 2004). Primary antibodies used in the immunoblotting experiments were mouse monoclonal anti-TIF2 (BD Biosciences, San Jose, CA) and mouse monoclonal anti-GAPDH (Chemicon International, Temecula, CA).

### Cell Proliferation Assay

Cell proliferation assays were performed using the xCELLigence system Real-Time Cell Analyzer (RTCA) DP instrument, as described by the manufacturer (Roche, Basel, Switzerland). MCF-7 cells were seeded in phenol red-free DMEM supplemented with 5% charcoal-stripped FBS for three days prior to the experiments. Background impedance was determined by incubating E-Plates with 100 mL cell medium for 30 minutes at 37°C and 5% CO_2_. 8×10^3^ control shRNA cells and SRC-2 shRNA cells were then seeded into individual wells of the E-plates and treated with 10 nM 17β-estradiol or Vehicle. To activate the PKA signalling pathway, the cells were treated with 8-CPT-cAMP (150 µM), IBMX (50 µM) and forskolin (10 µM). The plates containing the cells were then incubated at room temperature for 30 minutes before placing them into the RTCA-unit. Cell growth was measured for 120 hours by monitoring the impedance every 15 minutes. Impedance is represented by cell index (CI) and was calculated as follows: CI¼(Zi_Z0)/15W, where Zi is the impedance at an individual time-point, and Z0 is the background impedance. Average CI was calculated from a minimum of 2–4 wells per time-point and per experiment.

## Results

### Loss of SRC-2 in MCF-7 Breast Cancer Cells Induces Distinct Changes in the Global Gene Expression

**Figure 1:**
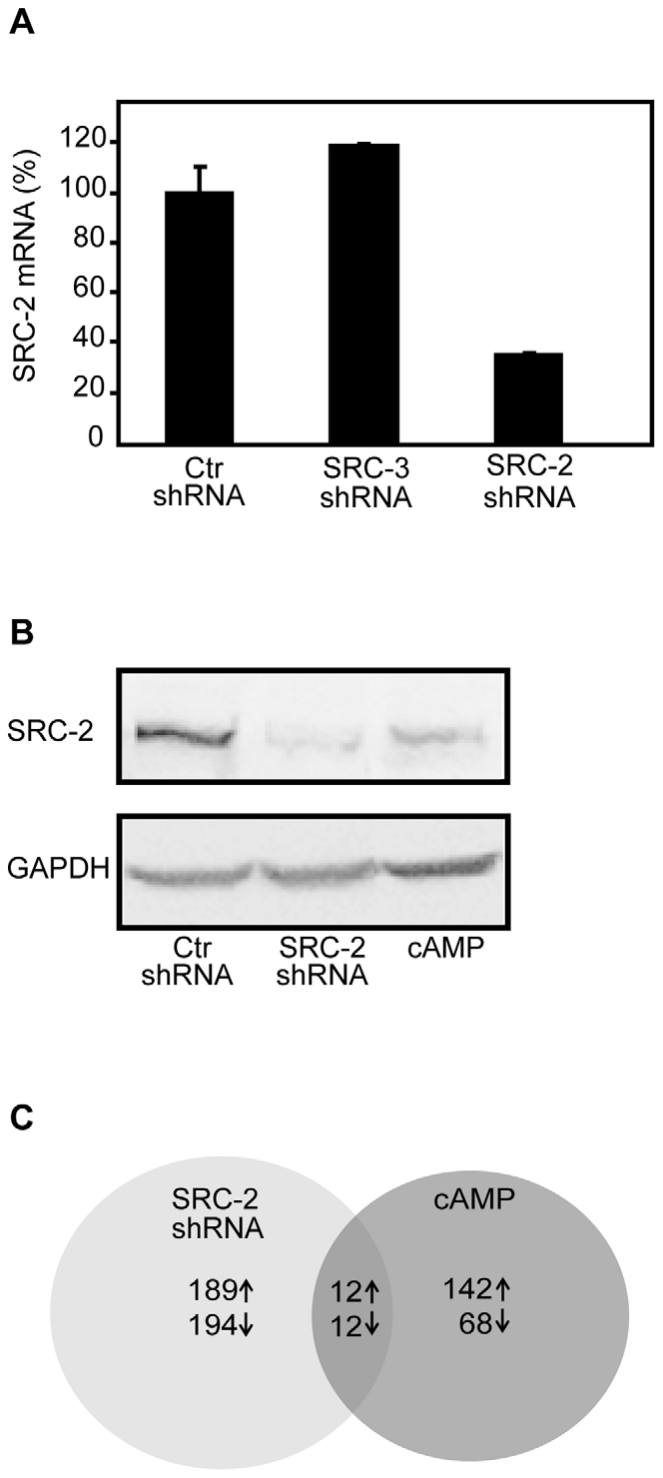
Downregulation of SRC-2 in MCF-7 cells induces distinct changes in global gene expression profiles. (A). Quantification of SRC-2 mRNA expression in shRNA lentivirus-infected MCF-7 cells. mRNA levels of SRC-2 in a MCF-7 cell line infected with shRNA targeting SRC-2 (SRC-2 shRNA) were compared to the expression in a control shRNA MCF-7 cell line (Ctr shRNA), and in a MCF-7 cell line transduced with shRNA lentivirus targeting SRC-3 (SRC-3 shRNA). The mRNA expression of SRC-2 is relative to TBP mRNA. The results are representative of at least three independent experiments. (B). Western blotting analyses of SRC-2-depleted MCF-7 cells. MCF-7 cells infected with shRNA lentivirus targeting SRC-2 (SRC-2 shRNA) or a negative control shRNA empty vector (Ctr shRNA), were grown in phenol red-free DMEM supplemented with charcoaled stripped FBS (5%) and 17β-estradiol (10 nM) for two days. The Ctr shRNA cells were then treated with either Vehicle or 8-CPT-cAMP (150 µM), IBMX (50 µM) and forskolin (10 µM) (cAMP) for 24 hours. Immunoblotting was performed with anti-TIF2 antibody and anti-GAPDH antibody. The results shown are representative of at least three independent experiments. (C). Microarray analyses of five RNA samples isolated from five individual cell samples of shRNA control MCF-7 cells (Ctr shRNA), SRC-2 KD MCF-7 cells (SRC-2 shRNA) and control shRNA cells treated with cAMP elevating agents, as described in A. A Venn diagram shows the number of individual and overlapping sets of genes differentially expressed after SRC-2 KD (SRC-2 shRNA) and after treatment with cAMP elevating agents. To examine which genes were similarly differentially expressed between the two treated groups when compared to control, a SAM analysis with overlapping genes was performed. The fold change cut-off value ≥1.5, and q-value = 0, was used to determine differentially expressed genes.

Previously we have shown that activation of PKA leads to increased ubiquitination and subsequent degradation of SRC-2, which in turn leads to inhibition of ERα transactivation function [35], [36]. Here we wanted to study the role of SRC-2 and the functional relevance of the PKA-mediated regulation of SRC-2 on gene expression in MCF-7 human breast cancer cells. Thus, we analysed global gene expression profiles of MCF-7 cells after either KD of SRC-2 or treatment with cAMP elevating agents. As shown in Figure 1A, there was approximately 65% reduction in SRC-2 mRNA expression after KD of SRC-2 (SRC-2 shRNA) compared to the control shRNA MCF-7 cells (Ctr shRNA), which was not found in a cell line expressing shRNA against SRC-3. Western blotting analyses confirmed successful KD of SRC-2 (Figure 1B). In order to activate PKA in the Ctr shRNA cells, they were treated with a cAMP analogue (8-CTP-cAMP) and cAMP elevating agents (IBMX and forskolin) for 24 hours. This time point was optimal to achieve degradation of SRC-2. As previously shown [36], [41], activation of PKA resulted in a significant reduction in SRC-2 protein level.

Five samples of RNA from each of the three different treatments (Ctr shRNA, SRC-2 shRNA and cAMP) were subjected to microarray analysis. Correspondence Analysis which displays the differences in global gene expression in a two-dimensional plot revealed a distinct separation between the three groups and no outliers, indicating differential expression of genes at the global level (Figure S1). Using a fold change ≥1.5 and a q-value = 0, a total number of 383 genes (194 downregulated and 189 upregulated) were differentially expressed in the SRC-2 KD cells (Tables S2 and S3), whereas the cAMP-treated cells showed 210 differentially expressed genes (68 downregulated and 142 upregulated) (Figure 1C), (Tables S4 and S5).

### Overlapping Gene Expression Changes Induced by SRC-2 Knockdown and cAMP

In order to study genes that could potentially be regulated through PKA-mediated downregulation of SRC-2, we searched for overlapping differentially expressed genes after SRC-2 KD or elevation of cAMP. As shown in the Venn diagram in Figure 1C, the expression of 12 genes decreased, whereas 12 genes showed increased expression both after SRC-2 KD or cAMP treatment (Table 1 and Table 2).

**Table 1:** Overlapping decreased expression changes relative to control induced by SRC-2 KD and cAMP in MCF-7 cells.

			Signal intensity			FC	
Gene	Definition		SRC-2 shRNA	cAMP	CtrshRNA	SRC-2 shRNA/Ctr shRNA	cAMP/Ctr shRNA
*AKR1B10*		aldo-keto reductase family 1, member B10 (aldose reductase)	255	257	507	−1.98	−1.98
*ARHGEF19*		Rho guanine nucleotide exchange factor (GEF) 19	420	415	632	−1.50	−1.53
*BCL11B*		B-cell CLL/lymphoma 11B (zinc finger protein),transcript variant 1	232	313	570	−2.46	−1.83
*CAV1*		caveolin 1, caveolae protein, 22kDa	3534	2946	5595	−1.59	−1.91
*EGR1*		early growth response 1	1159	1125	1799	−1.55	−1.62
*KLK5*		kallikrein-related peptidase 5, transcript variant 1	345	292	598	−1.74	−2.06
*KLK5*		kallikrein-related peptidase 5, transcript variant 2	244	226	416	−1.72	−1.84
*KRT1*		keratin 1 (epidermolytic hyperkeratosis)	143	118	229	−1.60	−1.94
*KRT5*		keratin 5	171	197	347	−2.03	−1.76
*KRTDAP*		keratinocyte differentiation-associated protein	173	283	571	−3.29	−2.02
*Protein S100-A9*		S100 calcium binding protein A9 (calgranulin B)	308	644	1345	−4.37	−2.11
*Protein S100-A8*		S100 calcium binding protein A8	242	530	1892	−7.82	−3.58
*TAGLN*		transgelin, transcript variant 2	257	350	797	−3.10	−2.28

Genes with FC ≥1.5 and q-value = 0 are shown in addition to their respective expression signals in the three microarray samples. cAMP, short-hairpin control MCF-7 cells treated with cAMP analogue (8-CPT-cAMP) and cAMP-elevating agents (IBMX and forskolin).

**Table 2:** Overlapping increased expression changes relative to control induced by SRC-2 KD and cAMP in MCF-7 cells.

		Signal Intensity			FC	
**Gene**	**Definition**	**SRC-2 shRNA**	**cAMP**	**Ctr shRNA**	**SRC-2 shRNA/Ctr shRNA**	**cAMP/Ctr shRNA**
*ADM*	adrenomedullin	336	270	176	1.90	1.54
*AKR1C2*	aldo-keto reductase family 1, member C2), transcript variant 1	10519	10085	6693	1.57	1.51
*BCAS1*	breast carcinoma amplified sequence 1	474	548	229	2.07	2.39
*CXCR4*	chemokine (C-X-C motif) receptor 4, transcript variant 2	411	285	187	2.19	1.51
*C9orf152*	chromosome 9 open reading frame 152	776	904	446	1.74	2.03
*FAM46A*	family with sequence similarity 46, member A	1647	3923	976	4.00	4.00
*LYPD6B*	(hypothetical protein LOC130576), LY6/PLAURdomain containing 6B	1361	1205	733	1.86	1.64
*NUCB2*	nucleobindin 2	625	778	413	1.50	1.87
*RET*	ret proto-oncogene, transcript variant 4	986	889	538	1.83	1.65
*RET*	ret proto-oncogene, transcript variant 2	1045	1077	524	1.99	2.04
*S100P*	S100 calcium binding protein P	3980	5622	1275	3.12	4.41
*TFF3*	trefoil factor 3 (intestinal)	17129	17494	10193	1.68	1.72
*TFPI*	tissue factor pathway inhibitor, transcript variant 2	537	606	355	1.10	1.19
*TFPI*	tissue factor pathway inhibitor, transcript variant 1	416	482	289	1.51	1.70

Genes with FC ≥1.5 and q-value = 0 are shown in addition to their respective expression signals in the three microarray samples. cAMP, short-hairpin control MCF-7 cells treated with cAMP analogue (8-CPT-cAMP) and cAMP-elevating agents (IBMX and forskolin).

**Figure 2:**
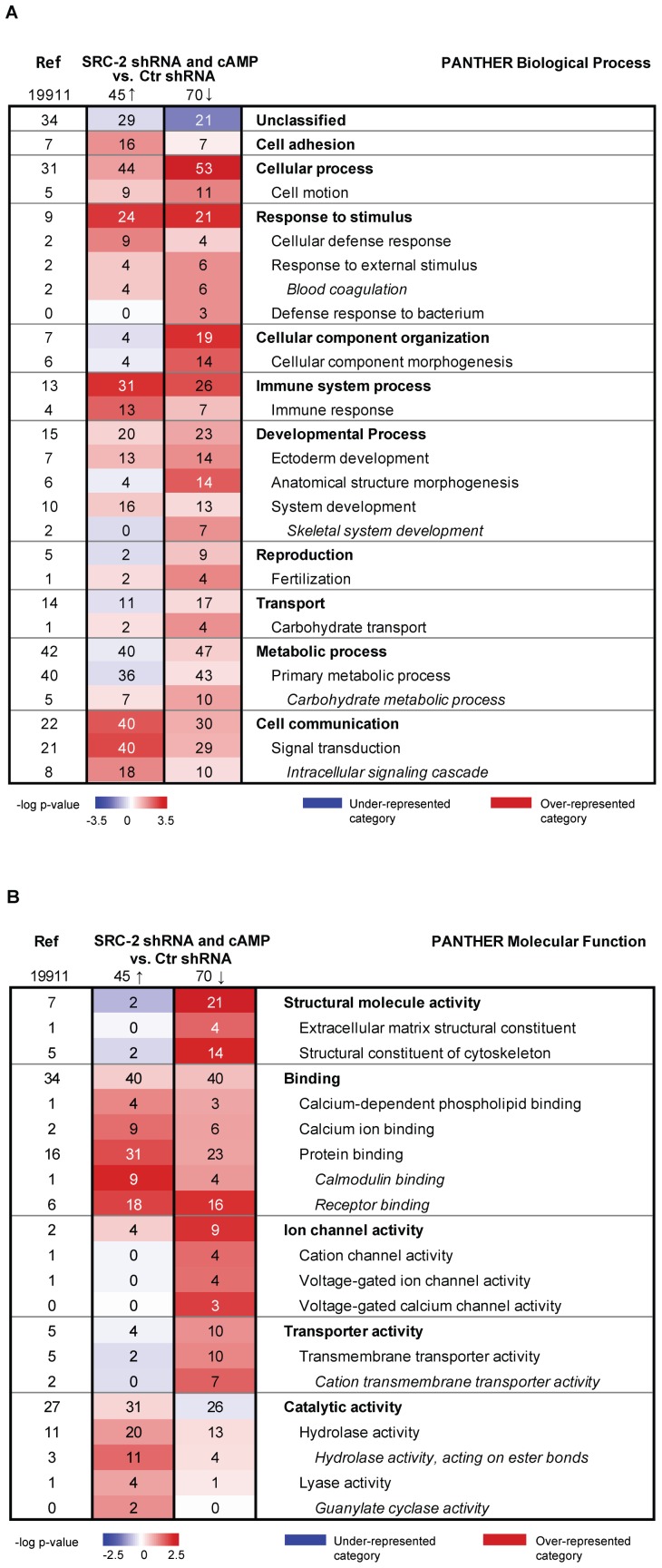
Functional categorization of differentially expressed overlapping genes. PANTHER was used to search for over-represented Biological Process categories (A) and Molecular Function categories (B) among the differentially expressed overlapping up-regulated genes after SRC-2 KD (SRC-2 shRNA) and after exposure of cAMP elevating agents, and among the differentially expressed overlapping downregulated genes from the same two samples (fold change ≥1.3, q-value = 0). Bonferroni correction for multiple testing was performed and a p-value of 0.05 was chosen as inclusion criterion for functional categories. The numbers in the figure are percentage numbers. I.e. the reference column at the left of the table displays the percentage of genes that belongs to a specific category when analysing the whole human NCBI genome (19,911 genes), e.g. 31% of the 19,911 genes belong to the “cellular process” category. The colour intensity scales are based on the statistical significance (-log p-value) of over- and under-represented PANTHER functional categories. Red colour displays over-represented categories and blue colour represents under-represented categories. A specific category will display red colour (over-representation) when there is a higher percentage of genes in the dataset (SRC-2 shRNA and cAMP vs. Ctr shRNA) compared to the percentage of genes in the reference column mapping to this specific category. E.g. 53% of the 70 downregulated genes map to the category “cellular process” while 31% of the reference genes map to this category, hence this category is over-represented among the downregulated genes and displays a red colour. The opposite principle will be true for the under-represented categories (blue colour). Ref, reference. Arrow up, up-regulated genes. Arrow down, downregulated genes.

To gain insight into the functions of the genes changed by both SRC-2 depletion and cAMP elevation, we performed gene ontology analyses of the overlapping genes using the PANTHER classification system. We observed that several of these genes were involved in the PANTHER Biological Process categories Cell cycle (e.g. *RET*, *S100P*, *KLK5*, *S100A8*, *S100A9,* and *EGR1*), Cell motion (e.g. *CXCR4 RET*, *TFF3*, *S100P*, *S100A8* and *S100A9*), Immune response (e.g. *CXCR4*, *S100P*, *S100A8*, *S100A9*, *TFF3*, *KLK5*, *RET* and *TFP1*), Signal transduction (e.g. *CXCR4*, *RET*, *TFF3*, *S100P*, *S100A8*, *S100A9* and *CAV1*) and Metabolic processes (e.g. *RET*, *TFP1*, *AKR1C2*, *KLK5*, *AKR1B10*, *BCL11B* and *EGR1*) (data not shown). In order to increase the number of overlapping genes to be included in the PANTHER analyses but simultaneously maintain a low chance of false positive hits, fold change cut-off value was lowered from 1.5 to 1.3 (q-value = 0). Our analyses revealed 124 overlapping genes by which 75 were decreased and 45 genes showed increased expression after SRC-2 KD and activation of PKA (Tables S6 and S7). As shown in Figure 2A, PANTHER functional analyses of the downregulated overlapping genes revealed a significant overrepresentation of the Biological Process categories referred to as Cellular process and Cellular component organization compared to control MCF-7 cells (Ctr shRNA). Analysing the overlapping upregulated genes we observed a clear overrepresentation of the Biological Process category Cell communication (Figure 2A). PANTHER analyses of Molecular Function categories showed that genes belonging to Structural molecular activity were overrepresented amongst the downregulated genes by SRC-2 KD- and cAMP (Figure 2B). This suggest that PKA stimulation and SRC-2 silencing in MCF-7 cells entails decreased expression of genes involved in cellular process as well as cellular component organization, whereas the expression of genes involved in cell communication was enhanced.

### Depletion of SRC-2 Downregulates Tumour Suppressor Genes and Upregulates Oncogenes

To confirm that gene expression changes observed in the microarray data were representative of the original samples, a subset of the differentially expressed overlapping genes, were selected for validation by qRT-PCR analysis (Table 3). Based on the fact that little is known about the role of SRC-2 in breast tumourigenesis, genes that have been associated with breast cancer were selected for validation by qRT-PCR. The five genes showing decreased expression after SRC-2 KD and PKA activation compared to control, *EGR1*, *TAGLN*, *CAV1*, *BCL11b* and *AKR1B10*, together with five overlapping genes showing increased expression, *RET* (tv2 and tv4), *BCAS1*, *TFF3*, *ADM*, *CXCR4*, were chosen for validation. *EGR1*, *CAV1*, *TAGLN* and *BCL11b* are estrogen-responsive genes described as tumour suppressors in breast cancer [42]–[45], whereas the upregulated genes are estrogen-responsive genes described as breast cancer oncogenes (*RET*, *BCAS1*, *TFF3*) [46]–[48] or known to stimulate breast cancer metastasis and progression (*CXCR4*, *ADM*) [49], [50]. We observed that the mRNA expression of these genes was reversed by overexpression of SRC-2 in MCF-7 cells (data not shown). As shown in Table 3, the qRT-PCR validation results were highly consistent with the microarray results, suggesting that SRC-2 is regulating estrogen-responsive genes involved in breast tumourigenesis. The results indicate that downregulation of SRC-2 mediates decreased expression of several tumour suppressor genes, whereas the expression of genes involved in oncogenesis were increased.

**Table 3:** Validation of selected genes by qRT-PCR.

	Microarray		Q-rt-PCR			
Gene	SRC-2 shRNA/Ctr shRNA	cAMP/Ctr shRNA	SRC-2 shRNA/Ctr shRNA	95% CI	cAMP/Ctr shRNA	95% CI
*ADM*	1.90	1.54	2.81	1.16–6.81	2.10	0.95–4.63
*BCAS1*	2.07	2.39	4.21	1.74–10.20	5.11	2.74–9.54
*CXCR4*	2.19	1.51	4.97	2.40–10.30	2.55	1.89–3.44
*RET (tv4)*	1.839	1.65	2.53	1.22–5.18	2.14	1.17–3.89
*RET (tv2)*	1.20	2.04	1.88	0.75–4.38	1.63	0.88–3.02
*TFF3*	1.68	1.72	2.46	1.06–5.72	2.44	1.46–3.78
*AKR1B10*	−1.98	−1.98	0.46	0.24–0.88	0.39	0.27–0.55
*BCL11B*	−2.45	−1.83	0.20	0.11–0.40	0.44	0.24–0.80
*CAV1*	−1.59	−1.91	0.57	0.33–0.99	0.45	0.37–0.56
*EGR1*	−1.55	−1.62	0.82	0.41–1.66	0.66	0.34–1.27
*TAGLN*	−3.10	−2.28	0.22	0.14–0.34	0.35	0.28–0.44

Fold change values from the microarray analyses compared to quantification of mRNA expression by qRT-PCR presented as geometric mean with 95% confidence intervals (CI), n = 5. cAMP, short-hairpin control MCF-7 cells treated with cAMP analogue (8-CPT-cAMP) and cAMP-elevating agents (IBMX and forskolin).

### Estrogen-responsive Genes Regulated Through cAMP/PKA-mediated Degradation of SRC-2

**Figure 3:**
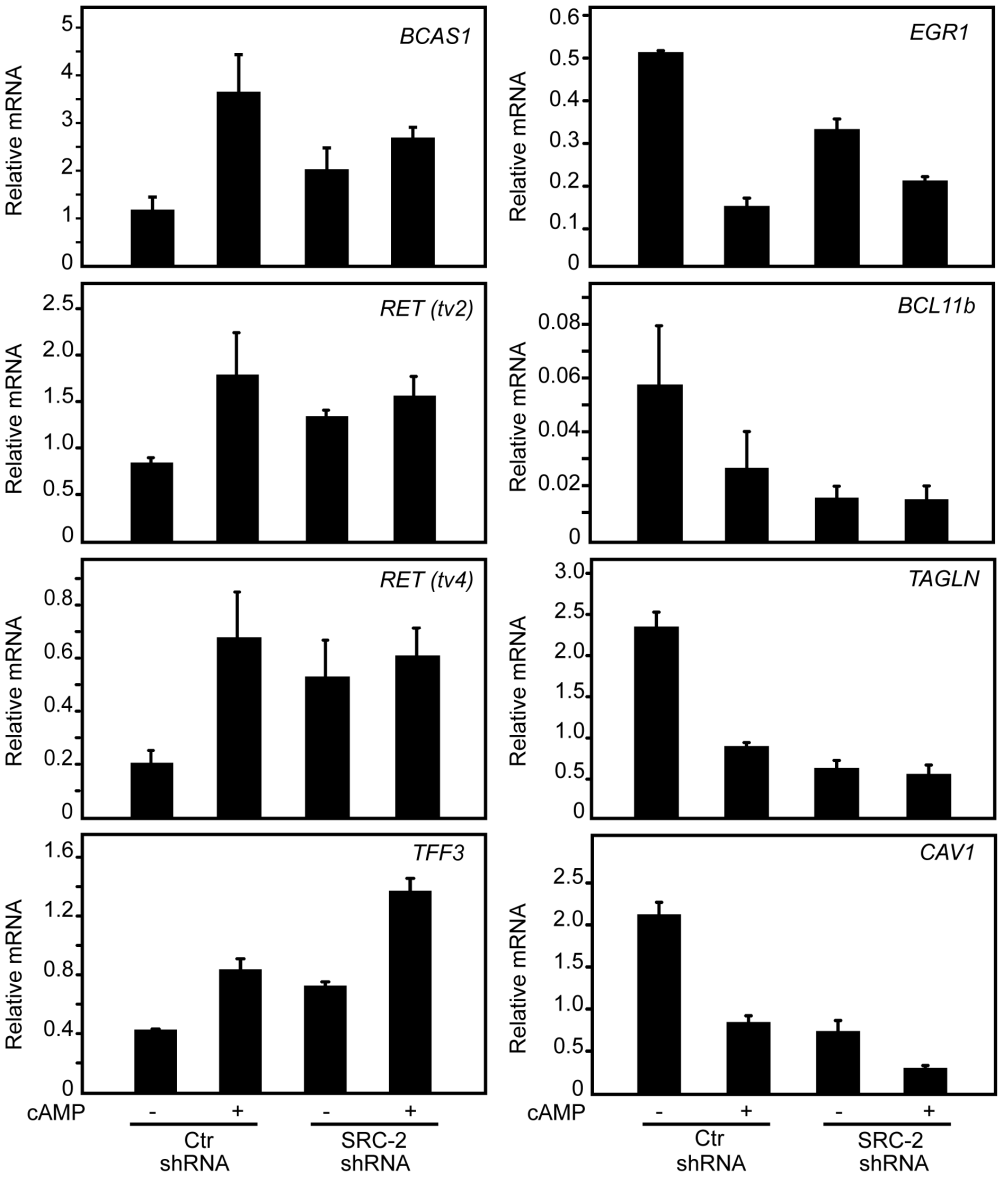
PKA-mediated downregulation of SRC-2 changes mRNA expression of ER-target genes. Control MCF-7 cells (Ctr shRNA) and SRC-2 KD MCF-7 cells (SRC-2 shRNA) were grown in phenol red-free DMEM supplemented with charcoaled stripped FBS (5%) and 17β-estradiol (10 nM) for two days. The two cell lines were then treated with Vehicle or 8-CPT-cAMP (150 µM), IBMX (50 µM) and forskolin (10 µM) (cAMP) for 24 hours. The mRNA expression of selected genes was measured by qRT-PCR. The expression level of each gene is relative to TBP mRNA. The results presents mean values ± SE obtained from four-six independent qRT-PCRs.

Next we wanted to verify whether the expression of the selected overlapping genes was regulated through PKA-mediated degradation of SRC-2. Thus, qRT-PCR was used to quantify the mRNA expression changes of selected genes in control MCF-7 cells and in SRC-2-depleted cells in response to treatment with PKA activating agents (Figure 3). The relative increase in mRNA expression of *BCAS1*, *RET* (tv2) and *RET* (tv4) due to PKA activation (cAMP) observed in the control shRNA cells was diminished or absent in the SRC-2 shRNA cells. In cells with reduced SRC-2 level, adding PKA activating agents did not result in any further increase in the expression of these three genes, suggesting that the cAMP effect is mediated via downregulation of SRC-2. In contrast, *TFF3* mRNA levels were increased by PKA in both cell lines, suggesting that this gene is also regulated by PKA via an SRC-2 independent pathway (Figure 3). The relative PKA-induced downregulation of *EGR1*, *BCL11b* and *TAGLN,* but not *CAV-1* was counteracted by SRC-2 KD. Together, these results suggested that expression of *BCAS1*, *RET*, *EGR1*, *BCL11b* and *TAGLN* are regulated through PKA-induced SRC-2 degradation (Figure 3), whereas PKA regulates the expression of *TFF3* and *CAV1* independently of SRC-2 degradation.

### Depletion of SRC-2 Stimulates Breast Cancer Cell Proliferation

**Figure 4:**
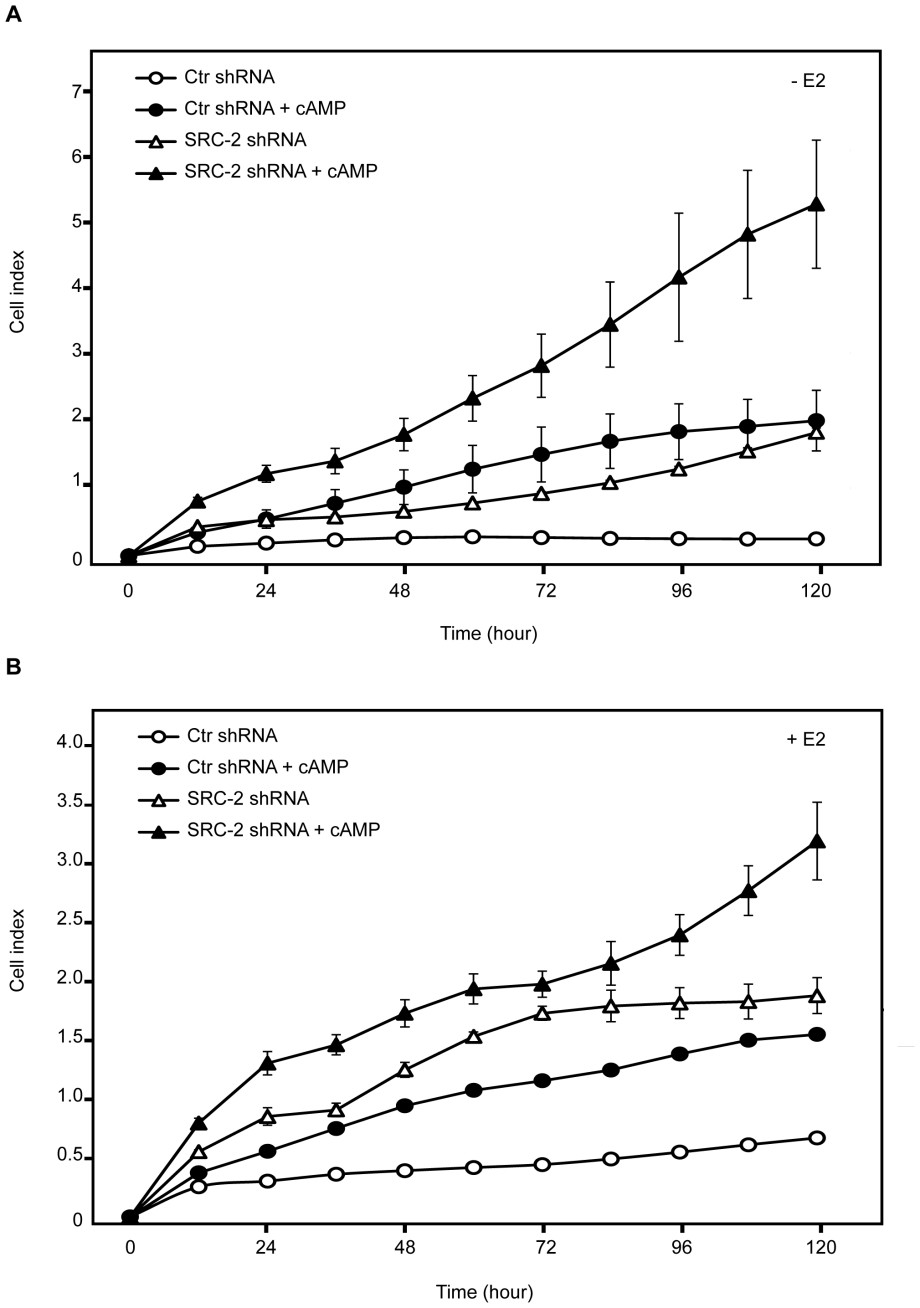
Downregulation of SRC-2 promotes proliferation of MCF-7 cells. MCF-7 cells with KD of SRC-2 (SRC-2 shRNA) and control shRNA MCF-7 cells (Ctr shRNA) grown in phenol red-free DMEM supplemented with charcoaled stripped FBS (5%) for three days, were seeded in E-Plate 16 and treated with either Vehicle or 8-CPT-cAMP (150 µM), IBMX (50 µM) and forskolin (10 µM) (cAMP), in the absence or presence of 17β-estradiol (E2, 10 nM) (A and B, respectively). Proliferation of the cells was measured by using the xCELLigence system monitoring cellular impedance continuously for 120 hours. The results are representative of at least three independent experiments.

Since our results indicated that KD of SRC-2 changes the expression of estrogen-responsive genes known to be involved in carcinogenesis, we wanted to examine whether KD of SRC-2 affected the real time growth of MCF-7 cells by using the xCELLigence System. We also examined the growth of control shRNA cells and SRC-2 shRNA cells treated with cAMP analogue and cAMP-elevating agents. The cell proliferation was monitored both in the absence and presence of 17β-estradiol. Interestingly, MCF-7 cells with reduced level of SRC-2 showed a significant increase in cell proliferation compared to the control shRNA cell line. This was observed both in the presence and absence of 17β-estradiol (Figures 4A and 4B). Moreover, we observed that MCF-7 cell growth increased significantly after treatment with the PKA-activating agents. The cAMP-stimulated growth was also observed in the SRC-2 KD cells (Figures 4A and 4B). MCF-7 cells treated with both SRC-2 shRNA and PKA-activating agents showed the most pronounced cell proliferation, suggesting that PKA has an effect on proliferation independent of SRC-2 degradation. Together, these data suggest that downregulation of SRC-2 induce proliferation of MCF-7 cells.

## Discussion

Several studies have examined how the different members of the SRC coactivator family promote carcinogenesis. The three SRCs are regulated by multiple upstream signalling pathways and changes in their protein levels or activity can effectively modulate gene expression. Unlike SRC-1 and SRC-3, which are overexpressed in different types of cancers, there are few reports regarding a role of SRC-2 in oncogenesis [51], [52]. In the present study, we explored the potential function of SRC-2 in MCF-7 breast cancer cells, and the role of PKA-mediated degradation of SRC-2 by characterization of the transcriptomes of SRC-2-depleted MCF-7 cells and of cells treated with PKA-activating agents. We observed that downregulation of SRC-2 induces significant changes in the expression of several estrogen-responsive genes involved in breast cancer progression. Consistent with these findings, we observed that depletion of SRC-2 in MCF-7 cells clearly stimulated proliferation of the cells. Together, the results suggest an antiproliferative role of SRC-2 in MCF-7 cells.

A recent study also demonstrated that low levels of SRC-2 expression in hepatocellular carcinoma patients were associated with poor prognosis, and RNAi-mediated knockdown of *NCOA2* in diethylnitrosamine-treated mice promoted liver tumourigenesis [53]. Moreover, it has been reported that enhanced expression of SRC-2 in malignant pleural mesothelioma (MPM) tumour cells is associated with improved prognosis [54]. SRC-2 is implicated in various cancers including colon, prostate, endometrial, liver, and astrocytic brain cancer [53], [55]–[58]. In breast tumour tissue, endocrine therapy has also been shown to induce the expression SRCs [59], [60]. Still, there are few reports regarding the contribution of SRC-2 in cell growth and its role in regulating genes involved in cell proliferation and cancer progression. Our findings suggest an inhibitory role of SRC-2 in breast tumourigenesis which differs from the established oncogenic function of two other SRC family members. A recent study demonstrated that SRC-3, but not SRC-2, is required for estradiol-dependent growth of breast cancer cells, which is in agreement with our observations [29]. Another report have shown that SRC-3, in contrast to SRC-2, stimulates proliferation of androgen-dependent and androgen-independent prostate cancer cell and tumour growth [61], indicating a different role of SRC-2 in these types of cancer. The reason for these apparent differences in growth regulation between SRC family members is not clear. However, it has been shown that the different SRCs have tissue-specific functions [62], as well as gene-specific roles in regulating estrogen-responsive genes in breast cancer cells [17], [29], [34]. It has also been demonstrated that recruitment of a particular member of the SRC family to a DNA-associated transcription factor is determined by the level of expression of that particular SRC, and PTMs may alter the availability of SRCs [33].

Activation of the PKA-signalling pathway specifically inhibits the coactivator function of SRC-2 in MCF-7 cells by promoting its ubiquitination and degradation and thereby reducing ERα transactivation function [35], [36]. In the present study we demonstrated that downregulation of SRC-2 by PKA modulates the expression of several estrogen-responsive genes involved in breast tumourigenesis, including *RET*, *BCAS1*, *EGR1*, *BCL11b* and *TAGLN*, indicating that PKA signalling through SRC-2 could be implicated in breast cancer progression. Moreover, functional analyses of differentially expressed genes after cAMP-treatment revealed enhanced expression of genes involved in cell cycle and cell signalling processes, as well as genes encoding signalling molecules such as growth factors (data not shown). In line with this, we observed enhanced proliferation of MCF-7 cells treated with PKA activating agents. Adding cAMP elevating agents to the SRC-2-silenced cells resulted in the most pronounced proliferation pattern. The gene expression and proliferation data suggest that PKA promotes tumourigenesis via both SRC-2-dependent- as well as -independent mechanisms. PKA is known to be implicated in initiation and progression of many tumours, but the mechanism by which PKA promote cancinogenesis is not clear [63]. The cAMP/PKA signalling pathway appears to have both proliferative and anti-proliferative effects on breast cancer cell growth [64]–[67], and various cAMP analogues have also been reported to exert different effects on breast cancer cell growth which may be due to differences in their mechanisms of action [67].

Even though SRC-2 is classified as a coactivator we observed that several genes also were up-regulated due to reduced SRC-2 expression, suggesting a repressive effect of SRC-2 on the expression of some estrogen-responsive genes in breast cancer cells. However, the mechanism by which estrogen represses gene expression is unknown. Studies have shown that SRC-2 in contrast to SRC-1 and SRC-3, possess a unique repression domain encompassing GRIP1 amino acids 767–1006 utilized in repression of GR-mediated inhibition of target gene expression [19]. It has been suggested that the enhancing- or repressive effect of SRC-2 on gene expression could be tissue specific and also to be dependent on the regulatory context of the target gene promoter. Since we observed that depletion of SRC-2 led to enhanced expression of several genes, including ERα-target genes, it is possible that SRC-2 may also function as a transcriptional corepressor of ERα in breast cancer cells.

Some studies have shown that the SRCs are able to compensate for the loss of individual SRC-member [68], [69]. However, a recent ChIP-sequencing mapping study of the global chromatin binding sites of individual SRCs in MCF-7 cells, revealed limited degree of redundancy between the different SRCs [34], which has also been seen in a study comparing the role of the three SRC family members on metabolism in different organs [70]. By analysing the expression of selected SRC-2- and PKA-regulated (overlapping) genes in SRC-3-depleted MCF-7 cells, we confirmed a specific regulation of these genes by SRC-2 (data not shown). In addition, analyses of transcription factor binding sites in the promoter regions of the selected overlapping genes showed enriched NR2F binding motifs in their promoters, which have shown by others to be exclusively found for SRC-2 target genes [34]. Thus, we believe that the oncogenic phenotype observed in the SRC-2-depleted cells is SRC-2 specific and not caused by compensatory effects of the other SRCs.

In summary, our data suggest that reduced levels of SRC-2 in breast cancer cells modulates the expression of estrogen-regulated genes leading to enhanced proliferation of breast cancer cells, suggesting that SRC-2 has antiproliferative properties in breast cancer cells.

## Supporting Information

Figure S1 **Correspondence analysis (CA) plot showing projection of microarray samples.** Ctr shRNA: pink squares, SRC-2 shRNA: green diamonds, cAMP: purple circles. The first principle component is shown on the x-axis and the second principle component is displayed on the y-axis. All three groups of samples are separated along the first principle component (22.706% component variance). The second principle component (15.192% component variance) separates the two groups of treated samples (SRC-2 shRNA and cAMP) from the control group sample (Ctr shRNA).
(TIF)

Table S1 **Primer sequences used for Q-rt-PCR.**
(DOC)

Table S2 **Downregulated differentially expressed genes induced by SRC-2 shRNA.** FC ≥1.5, q-value = 0.
(XLSX)

Table S3 **Upregulated differentially expressed genes induced by SRC-2 shRNA.** FC ≥1.5, q-value = 0.
(XLSX)

Table S4 **Downregulated differentially expressed genes induced by cAMP.** FC ≥1.5, q-value = 0.
(XLSX)

Table S5 **Upregulated differentially expressed genes induced by cAMP.** FC ≥1.5, q-value = 0.
(XLSX)

Table S6 **Overlapping downregulated differentially expressed genes induced by SRC-2 shRNA and cAMP.** FC ≥1.3, q-value = 0.
(XLSX)

Table S7 **Upregulated overlapping differentially expressed genes induced by SRC-2 shRNA and cAMP.** FC ≥1.3, q-value = 0.
(XLSX)

Abbreviations S1 (DOC)

## References

[1] Onate SA, Tsai SY, Tsai MJ, O’Malley BW (1995) Sequence and characterization of a coactivator for the steroid hormone receptor superfamily. Science 270: 1354–1357. 7481822 10.1126/science.270.5240.1354

[2] Hong H, Kohli K, Trivedi A, Johnson DL, Stallcup MR (1996) GRIP1, a novel mouse protein that serves as a transcriptional coactivator in yeast for the hormone binding domains of steroid receptors. Proc Natl Acad Sci U S A 93: 4948–4952. 8643509 10.1073/pnas.93.10.4948 PMC39385

[3] Voegel JJ, Heine MJ, Zechel C, Chambon P, Gronemeyer H (1996) TIF2, a 160 kDa transcriptional mediator for the ligand-dependent activation function AF-2 of nuclear receptors. Embo J 15: 3667–3675. 8670870 PMC452006

[4] Anzick SL, Kononen J, Walker RL, Azorsa DO, Tanner MM, et al (1997) AIB1, a steroid receptor coactivator amplified in breast and ovarian cancer. Science 277: 965–968. 9252329 10.1126/science.277.5328.965

[5] Chen H, Lin RJ, Schiltz RL, Chakravarti D, Nash A, et al (1997) Nuclear receptor coactivator ACTR is a novel histone acetyltransferase and forms a multimeric activation complex with P/CAF and CBP/p300. Cell 90: 569–580. 9267036 10.1016/s0092-8674(00)80516-4

[6] Li H, Gomes PJ, Chen JD (1997) RAC3, a steroid/nuclear receptor-associated coactivator that is related to SRC-1 and TIF2. Proc Natl Acad Sci U S A 94: 8479–8484. 9238002 10.1073/pnas.94.16.8479 PMC22964

[7] Takeshita A, Cardona GR, Koibuchi N, Suen CS, Chin WW (1997) TRAM-1, A novel 160-kDa thyroid hormone receptor activator molecule, exhibits distinct properties from steroid receptor coactivator-1. J Biol Chem 272: 27629–27634. 9346901 10.1074/jbc.272.44.27629

[8] Torchia J, Rose DW, Inostroza J, Kamei Y, Westin S, et al (1997) The transcriptional co-activator p/CIP binds CBP and mediates nuclear-receptor function. Nature 387: 677–684. 9192892 10.1038/42652

[9] Gehin M, Mark M, Dennefeld C, Dierich A, Gronemeyer H, et al (2002) The function of TIF2/GRIP1 in mouse reproduction is distinct from those of SRC-1 and p/CIP. Mol Cell Biol 22: 5923–5937. 12138202 10.1128/MCB.22.16.5923-5937.2002 PMC133972

[10] Mukherjee A, Amato P, Craig-Allred D, DeMayo FJ, O’Malley BW, et al.. (2007) Steroid receptor coactivator 2: an essential coregulator of progestin-induced uterine and mammary morphogenesis. Ernst Schering Found Symp Proc: 55–76. 10.1007/2789_2007_057 18540568

[11] Jeong JW, Kwak I, Lee KY, White LD, Wang XP, et al (2006) The genomic analysis of the impact of steroid receptor coactivators ablation on hepatic metabolism. Mol Endocrinol 20: 1138–1152. 16423883 10.1210/me.2005-0407

[12] Picard F, Gehin M, Annicotte J, Rocchi S, Champy MF, et al (2002) SRC-1 and TIF2 control energy balance between white and brown adipose tissues. Cell 111: 931–941. 12507421 10.1016/s0092-8674(02)01169-8

[13] Modder UI, Monroe DG, Fraser DG, Spelsberg TC, Rosen CJ, et al (2009) Skeletal consequences of deletion of steroid receptor coactivator-2/transcription intermediary factor-2. J Biol Chem 284: 18767–18777. 19423703 10.1074/jbc.M109.000836 PMC2707192

[14] Reineke EL, York B, Stashi E, Chen X, Tsimelzon A, et al (2012) SRC-2 Coactivator Deficiency Decreases Functional Reserve in Response to Pressure Overload of Mouse Heart. PLoS One 7: e53395. 23300926 10.1371/journal.pone.0053395 PMC3534027

[15] Jeong JW, Lee KY, Han SJ, Aronow BJ, Lydon JP, et al (2007) The p160 steroid receptor coactivator 2, SRC-2, regulates murine endometrial function and regulates progesterone-independent and -dependent gene expression. Endocrinology 148: 4238–4250. 17556502 10.1210/en.2007-0122

[16] Li X, Wong J, Tsai SY, Tsai MJ, O’Malley BW (2003) Progesterone and glucocorticoid receptors recruit distinct coactivator complexes and promote distinct patterns of local chromatin modification. Mol Cell Biol 23: 3763–3773. 12748280 10.1128/MCB.23.11.3763-3773.2003 PMC155204

[17] Won Jeong K, Chodankar R, Purcell DJ, Bittencourt D, Stallcup MR (2012) Gene-specific patterns of coregulator requirements by estrogen receptor-alpha in breast cancer cells. Mol Endocrinol 26: 955–966. 22543272 10.1210/me.2012-1066 PMC3355545

[18] Li S, Shang Y (2007) Regulation of SRC family coactivators by post-translational modifications. Cell Signal 19: 1101–1112. 17368849 10.1016/j.cellsig.2007.02.002

[19] Rogatsky I, Luecke HF, Leitman DC, Yamamoto KR (2002) Alternate surfaces of transcriptional coregulator GRIP1 function in different glucocorticoid receptor activation and repression contexts. Proc Natl Acad Sci U S A 99: 16701–16706. 12481024 10.1073/pnas.262671599 PMC139207

[20] Cvoro A, Tzagarakis-Foster C, Tatomer D, Paruthiyil S, Fox MS, et al (2006) Distinct roles of unliganded and liganded estrogen receptors in transcriptional repression. Mol Cell 21: 555–564. 16483936 10.1016/j.molcel.2006.01.014

[21] Rogatsky I, Zarember KA, Yamamoto KR (2001) Factor recruitment and TIF2/GRIP1 corepressor activity at a collagenase-3 response element that mediates regulation by phorbol esters and hormones. EMBO J 20: 6071–6083. 11689447 10.1093/emboj/20.21.6071 PMC125702

[22] Uhlenhaut NH, Barish GD, Yu RT, Downes M, Karunasiri M, et al (2013) Insights into negative regulation by the glucocorticoid receptor from genome-wide profiling of inflammatory cistromes. Mol Cell 49: 158–171. 23159735 10.1016/j.molcel.2012.10.013 PMC3640846

[23] Fleming FJ, Myers E, Kelly G, Crotty TB, McDermott EW, et al (2004) Expression of SRC-1, AIB1, and PEA3 in HER2 mediated endocrine resistant breast cancer; a predictive role for SRC-1. J Clin Pathol 57: 1069–1074. 15452162 10.1136/jcp.2004.016733 PMC1770462

[24] Murphy LC, Simon SL, Parkes A, Leygue E, Dotzlaw H, et al (2000) Altered expression of estrogen receptor coregulators during human breast tumorigenesis. Cancer Res 60: 6266–6271. 11103781

[25] Myers E, Fleming FJ, Crotty TB, Kelly G, McDermott EW, et al (2004) Inverse relationship between ER-beta and SRC-1 predicts outcome in endocrine-resistant breast cancer. Br J Cancer 91: 1687–1693. 15477868 10.1038/sj.bjc.6602156 PMC2409954

[26] Qin L, Liao L, Redmond A, Young L, Yuan Y, et al (2008) The AIB1 oncogene promotes breast cancer metastasis by activation of PEA3-mediated matrix metalloproteinase 2 (MMP2) and MMP9 expression. Mol Cell Biol 28: 5937–5950. 18644862 10.1128/MCB.00579-08 PMC2547002

[27] Qin L, Liu Z, Chen H, Xu J (2009) The steroid receptor coactivator-1 regulates twist expression and promotes breast cancer metastasis. Cancer Res 69: 3819–3827. 19383905 10.1158/0008-5472.CAN-08-4389 PMC2911143

[28] Zhao C, Yasui K, Lee CJ, Kurioka H, Hosokawa Y, et al (2003) Elevated expression levels of NCOA3, TOP1, and TFAP2C in breast tumors as predictors of poor prognosis. Cancer 98: 18–23. 12833450 10.1002/cncr.11482

[29] Karmakar S, Foster EA, Smith CL (2009) Unique roles of p160 coactivators for regulation of breast cancer cell proliferation and estrogen receptor-alpha transcriptional activity. Endocrinology 150: 1588–1596. 19095746 10.1210/en.2008-1001 PMC2659266

[30] Planas-Silva MD, Shang Y, Donaher JL, Brown M, Weinberg RA (2001) AIB1 enhances estrogen-dependent induction of cyclin D1 expression. Cancer Res 61: 3858–3862. 11358796

[31] List HJ, Lauritsen KJ, Reiter R, Powers C, Wellstein A, et al (2001) Ribozyme targeting demonstrates that the nuclear receptor coactivator AIB1 is a rate-limiting factor for estrogen-dependent growth of human MCF-7 breast cancer cells. J Biol Chem 276: 23763–23768. 11328819 10.1074/jbc.M102397200

[32] Xu J, Wu RC, O’Malley BW (2009) Normal and cancer-related functions of the p160 steroid receptor co-activator (SRC) family. Nat Rev Cancer 9: 615–630. 19701241 10.1038/nrc2695 PMC2908510

[33] Zhang H, Yi X, Sun X, Yin N, Shi B, et al (2004) Differential gene regulation by the SRC family of coactivators. Genes Dev 18: 1753–1765. 15256502 10.1101/gad.1194704 PMC478195

[34] Zwart W, Theodorou V, Kok M, Canisius S, Linn S, et al (2011) Oestrogen receptor-co-factor-chromatin specificity in the transcriptional regulation of breast cancer. EMBO J 30: 4764–4776. 22002538 10.1038/emboj.2011.368 PMC3243612

[35] Fenne IS, Hoang T, Hauglid M, Sagen JV, Lien EA, et al (2008) Recruitment of coactivator glucocorticoid receptor interacting protein 1 to an estrogen receptor transcription complex is regulated by the 3′,5′-cyclic adenosine 5’-monophosphate-dependent protein kinase. Endocrinology 149: 4336–4345. 18499756 10.1210/en.2008-0037

[36] Hoang T, Fenne IS, Cook C, Borud B, Bakke M, et al (2004) cAMP-dependent protein kinase regulates ubiquitin-proteasome-mediated degradation and subcellular localization of the nuclear receptor coactivator GRIP1. J Biol Chem 279: 49120–49130. 15347661 10.1074/jbc.M409746200

[37] Hoang T, Fenne IS, Madsen A, Bozickovic O, Johannessen M, et al (2013) cAMP Response Element-Binding Protein Interacts With and Stimulates the Proteasomal Degradation of the Nuclear Receptor Coactivator GRIP1. Endocrinology 154: 1513–1527. 23462962 10.1210/en.2012-2049 PMC5393311

[38] Bolstad BM, Irizarry RA, Astrand M, Speed TP (2003) A comparison of normalization methods for high density oligonucleotide array data based on variance and bias. Bioinformatics 19: 185–193. 12538238 10.1093/bioinformatics/19.2.185

[39] Fellenberg K, Hauser NC, Brors B, Neutzner A, Hoheisel JD, et al (2001) Correspondence analysis applied to microarray data. Proc Natl Acad Sci U S A 98: 10781–10786. 11535808 10.1073/pnas.181597298 PMC58552

[40] Tusher VG, Tibshirani R, Chu G (2001) Significance analysis of microarrays applied to the ionizing radiation response. Proc Natl Acad Sci U S A 98: 5116–5121. 11309499 10.1073/pnas.091062498 PMC33173

[41] Borud B, Hoang T, Bakke M, Jacob AL, Lund J, et al (2002) The nuclear receptor coactivators p300/CBP/cointegrator-associated protein (p/CIP) and transcription intermediary factor 2 (TIF2) differentially regulate PKA-stimulated transcriptional activity of steroidogenic factor 1. Mol Endocrinol 16: 757–773. 11923473 10.1210/mend.16.4.0799

[42] Assinder SJ, Stanton JA, Prasad PD (2009) Transgelin: an actin-binding protein and tumour suppressor. Int J Biochem Cell Biol 41: 482–486. 18378184 10.1016/j.biocel.2008.02.011

[43] Baron V, Adamson ED, Calogero A, Ragona G, Mercola D (2006) The transcription factor Egr1 is a direct regulator of multiple tumor suppressors including TGFbeta1, PTEN, p53, and fibronectin. Cancer Gene Ther 13: 115–124. 16138117 10.1038/sj.cgt.7700896 PMC2455793

[44] Kamimura K, Mishima Y, Obata M, Endo T, Aoyagi Y, et al (2007) Lack of Bcl11b tumor suppressor results in vulnerability to DNA replication stress and damages. Oncogene 26: 5840–5850. 17369851 10.1038/sj.onc.1210388

[45] Hino M, Doihara H, Kobayashi K, Aoe M, Shimizu N (2003) Caveolin-1 as tumor suppressor gene in breast cancer. Surg Today 33: 486–490. 14506991 10.1007/s10595-002-2538-4

[46] Collins C, Rommens JM, Kowbel D, Godfrey T, Tanner M, et al (1998) Positional cloning of ZNF217 and NABC1: genes amplified at 20q13.2 and overexpressed in breast carcinoma. Proc Natl Acad Sci U S A 95: 8703–8708. 9671742 10.1073/pnas.95.15.8703 PMC21140

[47] Eng C (1999) RET proto-oncogene in the development of human cancer. J Clin Oncol 17: 380–393. 10458257 10.1200/JCO.1999.17.1.380

[48] Morandi A, Plaza-Menacho I, Isacke CM (2011) RET in breast cancer: functional and therapeutic implications. Trends Mol Med 17: 149–157. 21251878 10.1016/j.molmed.2010.12.007

[49] Liang Z, Yoon Y, Votaw J, Goodman MM, Williams L, et al (2005) Silencing of CXCR4 blocks breast cancer metastasis. Cancer Res 65: 967–971. 15705897 PMC3734941

[50] Martinez A, Vos M, Guedez L, Kaur G, Chen Z, et al (2002) The effects of adrenomedullin overexpression in breast tumor cells. J Natl Cancer Inst 94: 1226–1237. 12189226 10.1093/jnci/94.16.1226

[51] Walsh CA, Qin L, Tien JC, Young LS, Xu J (2012) The function of steroid receptor coactivator-1 in normal tissues and cancer. Int J Biol Sci 8: 470–485. 22419892 10.7150/ijbs.4125 PMC3303173

[52] Ma G, Ren Y, Wang K, He J (2011) SRC-3 has a role in cancer other than as a nuclear receptor coactivator. Int J Biol Sci 7: 664–672. 21647249 10.7150/ijbs.7.664 PMC3107475

[53] O’Donnell KA, Keng VW, York B, Reineke EL, Seo D, et al (2012) A Sleeping Beauty mutagenesis screen reveals a tumor suppressor role for Ncoa2/Src-2 in liver cancer. Proc Natl Acad Sci U S A 109: E1377–1386. 22556267 10.1073/pnas.1115433109 PMC3361419

[54] Jennings CJ, O’Grady A, Cummins R, Murer B, Al-Alawi M, et al (2012) Sustained expression of steroid receptor coactivator SRC-2/TIF-2 is associated with better prognosis in malignant pleural mesothelioma. J Thorac Oncol 7: 243–248. 22011668 10.1097/JTO.0b013e31822f6544

[55] Grivas PD, Tzelepi V, Sotiropoulou-Bonikou G, Kefalopoulou Z, Papavassiliou AG, et al (2009) Estrogen receptor alpha/beta, AIB1, and TIF2 in colorectal carcinogenesis: do coregulators have prognostic significance? Int J Colorectal Dis 24: 613–622. 19198856 10.1007/s00384-009-0647-9

[56] Gregory CW, He B, Johnson RT, Ford OH, Mohler JL, et al (2001) A mechanism for androgen receptor-mediated prostate cancer recurrence after androgen deprivation therapy. Cancer Res 61: 4315–4319. 11389051

[57] Kershah SM, Desouki MM, Koterba KL, Rowan BG (2004) Expression of estrogen receptor coregulators in normal and malignant human endometrium. Gynecol Oncol 92: 304–313. 14751175 10.1016/j.ygyno.2003.10.007

[58] Kefalopoulou Z, Tzelepi V, Zolota V, Grivas PD, Christopoulos C, et al (2012) Prognostic value of novel biomarkers in astrocytic brain tumors: nuclear receptor co-regulators AIB1, TIF2, and PELP1 are associated with high tumor grade and worse patient prognosis. J Neurooncol 106: 23–31. 21735116 10.1007/s11060-011-0637-y

[59] Haugan Moi LL, Hauglid Flageng M, Gandini S, Guerrieri-Gonzaga A, Bonanni B, et al (2010) Effect of low-dose tamoxifen on steroid receptor coactivator 3/amplified in breast cancer 1 in normal and malignant human breast tissue. Clin Cancer Res 16: 2176–2186. 20332317 10.1158/1078-0432.CCR-09-1859

[60] Flageng MH, Moi LL, Dixon JM, Geisler J, Lien EA, et al (2009) Nuclear receptor co-activators and HER-2/neu are upregulated in breast cancer patients during neo-adjuvant treatment with aromatase inhibitors. Br J Cancer 101: 1253–1260. 19755984 10.1038/sj.bjc.6605324 PMC2768454

[61] Zou JX, Zhong Z, Shi XB, Tepper CG, deVere White RW, et al (2006) ACTR/AIB1/SRC-3 and androgen receptor control prostate cancer cell proliferation and tumor growth through direct control of cell cycle genes. Prostate 66: 1474–1486. 16921507 10.1002/pros.20477

[62] Han SJ, DeMayo FJ, Xu J, Tsai SY, Tsai MJ, et al (2006) Steroid receptor coactivator (SRC)-1 and SRC-3 differentially modulate tissue-specific activation functions of the progesterone receptor. Mol Endocrinol 20: 45–55. 16141356 10.1210/me.2005-0310

[63] Mucignat-Caretta ACaC (2011) Protein Kinase A in Cancer. Cancers. 913–926. 10.3390/cancers3010913 PMC3756396 24212646

[64] Al-Dhaheri MH, Rowan BG (2006) PKA exhibits selective modulation of estradiol dependent transcription in breast cancer cells that is associated with decreased ligand binding, altered ER{alpha} promoter interaction and changes in receptor phosphorylation. Mol Endocrinol. 10.1210/me.2006-0059 17068199

[65] Dudek P, Picard D (2008) Genomics of signaling crosstalk of estrogen receptor alpha in breast cancer cells. PLoS One 3: e1859. 18365014 10.1371/journal.pone.0001859 PMC2268000

[66] Kung W, Roos W, Eppenberger U (1983) Growth stimulation of human breast cancer MCF-7 cells by dibutyryl cyclic AMP. Cell Biol Int Rep 7: 345–351. 6303604 10.1016/0309-1651(83)90074-7

[67] Lamb D, Steinberg RA (2002) Anti-proliferative effects of 8-chloro-cAMP and other cAMP analogs are unrelated to their effects on protein kinase A regulatory subunit expression. J Cell Physiol 192: 216–224. 12115728 10.1002/jcp.10131

[68] Xu J, Li Q (2003) Review of the in vivo functions of the p160 steroid receptor coactivator family. Mol Endocrinol 17: 1681–1692. 12805412 10.1210/me.2003-0116

[69] Tien JC, Zhou S, Xu J (2009) The role of SRC-1 in murine prostate cancinogenesis is nonessential due to a possible compensation of SRC-3/AIB1 overexpression. Int J Biol Sci 5: 256–264. 19305643 10.7150/ijbs.5.256 PMC2659009

[70] York B, Sagen JV, Tsimelzon A, Louet JF, Chopra AR, et al (2013) Research resource: tissue- and pathway-specific metabolomic profiles of the steroid receptor coactivator (SRC) family. Mol Endocrinol 27: 366–380. 23315938 10.1210/me.2012-1324 PMC3683811

