# Supplementary figures and images for: Downregulation of Steroid Receptor Coactivator-2 Modulates Estrogen-Responsive Genes and Stimulates Proliferation of MCF-7 Breast Cancer Cells
Source: PLoS One. 2013 Jul 30;8(7):e70096. doi: 10.1371/journal.pone.0070096 (PMC3728357; doi:10.1371/journal.pone.0070096)

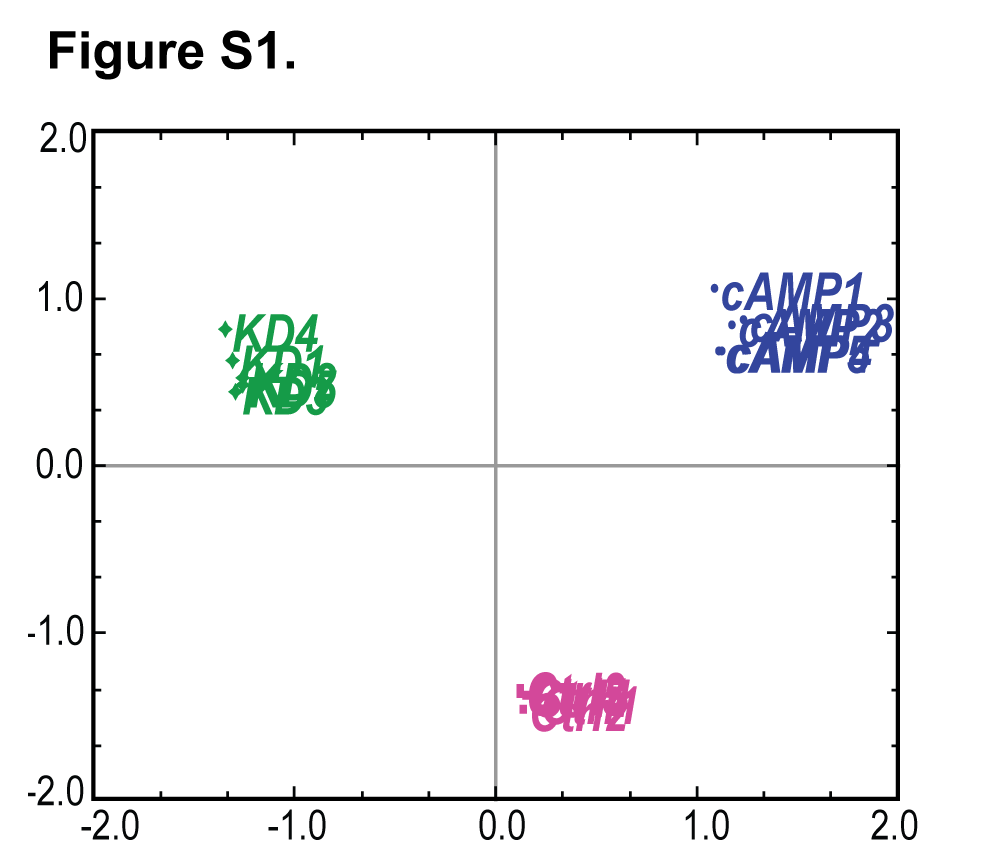

Supplement: Figure S1 — Correspondence analysis (CA) plot showing projection of microarray samples. Ctr shRNA: pink squares, SRC-2 shRNA: green diamonds, cAMP: purple circles. The first principle component is shown on the x-axis and the second principle component is displayed on the y-axis. All three groups of samples are separated along the first principle component (22.706% component variance). The second principle component (15.192% component variance) separates the two groups of treated samples (SRC-2 shRNA and cAMP) from the control group sample (Ctr shRNA). (TIF) [file pone.0070096.s001.tif]
