# Supplementary material for: Downregulation of Steroid Receptor Coactivator-2 Modulates Estrogen-Responsive Genes and Stimulates Proliferation of MCF-7 Breast Cancer Cells
Source: PLoS One. 2013 Jul 30;8(7):e70096. doi: 10.1371/journal.pone.0070096 (PMC3728357; doi:10.1371/journal.pone.0070096)
Supplement: Table S1 — Primer sequences used for Q-rt-PCR. (DOC) [file pone.0070096.s002.doc]

**Supporting Information Materials and Methods**

**Table S1.** Primer sequences used for Q-rt-PCR

| **Gene** | **Forward (5'-3')** | **Reverse (5'-3')** |
| --- | --- | --- |
| *RET (transcript variant 4)* | ctccgtggatgccttcaa | ccaagttcttccgagggaat |
| *RET (transcript variant 2)* | ctcccttccacatggattga | tcagctctcgtgagtggtaca |
| *TFF3* | gctgctgctttgactccag | ggaggtgcctcagaaggtg |
| *BCAS1* | agagcatcaagacaaggtgga | ctttttccttgccgtcaact |
| *ADM* | gcctgcccagacccttat | gtagcgcttgactcggatg |
| *CXCR4 (transcript variant 2)* | attgggatcagcatcgactc | caaactcacacccttgcttg |
| *BCL11B (transcritp variant 1)* | caagcaggagaacattgcag | gtgatcacggatgagtgagg |
| *EGR1* | agggacagcgctccagta | ggatcatgggaacctggaa |
| *CAV1* | tctctgtgggctggcagt | catgctgacctcattcagttg |
| *TAGLN (transcript variant 2)* | gtccgaacccagacacaagt | gccatgtctggggaaaga |
| *AKR1B10* | cccaggttctgatccgttt | tcactcaatttaaagtcaaagacctg |
| *SRC-2* | gagtaccaacggcacc | aggttgctgaccgtag |
