# Supplementary material for: Downregulation of Steroid Receptor Coactivator-2 Modulates Estrogen-Responsive Genes and Stimulates Proliferation of MCF-7 Breast Cancer Cells
Source: PLoS One. 2013 Jul 30;8(7):e70096. doi: 10.1371/journal.pone.0070096 (PMC3728357; doi:10.1371/journal.pone.0070096)
Supplement: Abbreviations S1 — (DOC) [file pone.0070096.s009.doc]

Abbreviations^1^ The abbreviations used are:

8-CPT-cAMP, 8-parachlorophenylthio-cyclic adenosine monophosphate;

AIB1, amplified in breast 1;

CI, cell index;

DMEM, Dulbecco’s modified eagle’s Medium;

ERα, estrogen receptor alpha;

FBS, fetal bovine serum;

GR, glucocorticoid receptor;

GRIP1, glucocorticoid receptor-interacting protein 1;

IBMX, 3-isobutyl-1-methylxanthine;

KD, knock down;

MCF-7, michigan cancer foundation-7;

NCOA2, nuclear receptor coactivator 2;

NR, nuclear receptor;

PANTHER, Protein Analysis Through Evolutionary Relationships;

PKA, protein kinase A;

PTMs, post translational modification;

qRT-PCR, quantitative real time- polymerase chain reaction;

Sh, short hairpin;

SRC, steroid receptor coactivator;

TIF2, transcription intermediary factor 2;
